# A Simplified, Graded, Electrodiagnostic Criterion for Guillain-Barré Syndrome That Incorporates Sensory Nerve Conduction Studies

**DOI:** 10.1038/s41598-019-44090-w

**Published:** 2019-05-22

**Authors:** Thirugnanam Umapathi, Christen Sheng Jie Lim, Brandon Chin Jie Ng, Eunice Jin Hui Goh, O. Ohnmar

**Affiliations:** 1 National Neuroscience Institute, Department of Neurology, Singapore, Singapore; 2 National University Hospital, University Medicine Cluster, Singapore, Singapore; 3 National University of Singapore, Yong Loo Lin School of Medicine, Singapore, Singapore; 4 Yangon General Hospital, Yangon, Myanmar

**Keywords:** Neurology, Peripheral neuropathies

## Abstract

Traditional electrodiagnostic (EDX) criteria for Guillain-Barré Syndrome (GBS), e.g. those delineated by Ho *et al*. and Hadden *et al*., rely on motor nerve conduction studies (NCS), and focus on differentiating GBS subtypes instead of the accurate diagnosis of GBS. Sensory studies, including the sural-sparing pattern, are not routinely used in GBS EDX. We studied the utility of a simplified criterion that utilizes sensory NCS. Motor and sensory NCS abnormalities were defined by comparing against age and height adjusted norms derived from 245 controls. We considered the sural-sparing pattern a positive diagnostic feature. We analyzed 109 prospectively validated GBS patients and graded them as “Definite”, “Probable” and “Possible” based on the number of motor and sensory abnormalities detected. Using proposed EDX criteria, 35.8%, 43.1%, 11.9% of all GBS patients were considered “Definite”, “Probable” or “Possible” respectively; whereas traditional EDX criteria only diagnosed 49.5% of cases. 27.5%, 35.3% and 21.6% of patients with the Miller-Fisher Syndrome (MFS) subtype of GBS were considered “Definite”, “Probable” or “Possible” respectively. In comparison, traditional criteria only detected 15.7% of cases. Our proposed EDX criterion, that includes sensory NCS, improves and grades the diagnostic certainty of GBS, especially MFS.

## Introduction

Commonly used electrodiagnostic (EDX) criteria for Guillain-Barré Syndrome (GBS)^1–4^ rely solely on motor nerve conduction studies (NCS), and focus on differentiating axonal from demyelinating subtypes. 60–70% of GBS patients have abnormal sensory NCS^5–9^. In Miller-Fisher Syndrome (MFS) sensory NCS abnormalities are more common than motor^10,11^. Furthermore, the preferential sparing of sural sensory nerve action potential (SNAP) is seen in almost half of GBS patients^5,8,9^ and is present in both demyelinating and non-demyelinating subtypes, including MFS^12^. It has been shown to be fairly specific for GBS and reliably distinguishes it from mimics^13^. We therefore hypothesized that we could improve GBS EDX by adding sensory NCS, in particular the sural-sparing pattern, and simplifying the motor NCS criteria by removing pre-determined thresholds for NCS abnormalities^2,3^ (e.g. an F-response latency more than 120% of the upper limit of normal) that were delineated for the purpose of distinguishing axonal and demyelinating pathology (Table 1). We believe these measures would improve GBS EDX, with regards to both sensitivity and specificity. It would also shift the emphasis to the primary aim of accurate, early electrodiagnosis of GBS rather than the secondary aim of subtyping axonal and demyelinating forms of GBS.

**Table 1:** Proposed EDX criterion for diagnosis of GBS.

Definite	Probable	Possible
2 abnormal motor nerves* AND the sural-sparing pattern**	2 abnormal motor nerves* AND either normal SNAP or a diffuse (non sural-sparing) decrease in SNAP** OR	1 abnormal motor nerve* with or without sensory nerve abnormalities OR
	1 abnormal motor nerve* AND the sural-sparing pattern	Normal motor nerve NCS with SNAP decrease** (either diffuse or in sural-sparing pattern)
	SNAP changes cannot be isolated to the sural nerve	

*Any of the following (norms must be age and height corrected, and NCS temperature controlled): Decreased CMAP, prolonged DML, decreased motor conduction velocity, temporal dispersion (increased duration of more than 30% compared to the distal), conduction block (decrease of more than 50% in proximal CMAP compared to distal without an increase on duration of more than 30%), prolonged F latency.

**Decrease in SNAP amplitude (norms must be age and height corrected, and temperature controlled).

## Results

### Cohort characteristics

One hundred and nine patients were studied. The mean, median ages were 48 and 47 years respectively (range 13 to 81). There were more males (male: female ratio 1.6). Fifty eight were GBS while 51 were MFS cases. The initial NCS was done at the following periods from onset of weakness:

- Within 1 week: 63 patients
- 8 to 14 days: 35 patients
- 14 to 21 days: 8 patients
- 21 to 28 days: 3 patients

The 3 patients who had initial studies more than 4 weeks after onset of symptoms, for various logistical reasons, were excluded.

### SNAP abnormalities in our cohort

Decreased SNAP amplitude was seen in 63 out of the 109 patients (58%). Of all patients with SNAP amplitude reductions, 71% demonstrated a sural-sparing pattern. Only 54 (50%) of the 109 patients had motor NCS abnormalities that satisfied traditional EDX criteria for GBS. Of the remaining half of patients, 33% showed reductions in SNAP. MFS/MFS-overlap cases accounted for 14 of these 18 patients. More importantly, 25% of those not satisfying the traditional EDX criteria for GBS had the sural-sparing pattern. Like in the study by Albers *et al*.^5^ isolated sural nerve sensory abnormality was rare; in our study population only 1 patient (0.9%) showed sural SNAP abnormality with no other abnormal sensory nerves.

### Application of modified GBS EDX criterion to the cohort

When using our proposed GBS EDX criterion, 39 (35.8%), 47 (43.1%) and 13 (11.9%) of all GBS patients were classified as “Definite”, “Probable” and “Possible” GBS respectively (total 96.6%; Table 2). Of the 10 (9.2%) patients not diagnosed with our criterion, 9 had a normal nerve conduction study and 1 had isolated sural SNAP abnormality. Traditional EDX criteria diagnosed 49.5% of the cases. In the GBS group excluding MFS, 25 (43.1%), 29 (50.0%) and 2 (3.5%) satisfied criteria for “Definite”, “Probable” and “Possible” GBS respectively (total 96.6%); an improvement from the 79.3% achieved by traditional criteria. Likewise, 14 (27.5%), 18 (35.3%) and 11 (21.6%) of the MFS group satisfied “Definite”, “Probable” and “Possible” criteria respectively (total 84.3%); a marked improvement from 15.7% diagnosed by the traditional criteria (Table 2).

**Table 2:** Comparison of diagnostic yield of traditional criteria against proposed criterion.

	GBS excluding MFS patients *(n* = 58)*	MFS *(n* = *51)*	Total *(n* = 109)*
TRADITIONAL CRITERIA	**46 (79.3%)**	**8 (15.7%)**	**54 (49.5%)**
PROPOSED CRITERION			
Total	**56 (96.6%)**	**43 (84.3%)**	**99 (90.8%)**
*Definite*	*25 (43*.*1%)*	*14 (27*.*5%)*	*39 (35*.*8%)*
*Probable*	*29 (50*.*0%)*	*18 (35*.*3%)*	*47 (43*.*1%)*
*Possible*	*2 (3*.*5%)*	*11 (21*.*6%)*	*13 (11*.*9%)*
*Normal NCS*	*1 (1*.*7%)*	*8 (15*.*7%)*	*9 (8*.*3%)*

*One patient had isolated sural SNAP abnormality and therefore was the only GBS case not categorised.

## Discussion

The various EDX criteria for GBS have been remarkable for:

- A focus on distinguishing axonal and demyelinating GBS rather than on the early identification of GBS in patients presenting with acute flaccid paralysis.
- The exclusion of sensory nerve conduction studies even though sensory abnormalities are common. Furthermore, as shown by Derksen *et al*., the sural-sparing pattern can offer specificity by helping distinguish GBS from its mimics^13^.
- Insensitivity in diagnosing MFS which, in some parts of the world, is as common as paralytic GBS.

We have found that the simplification of the motor criteria and the inclusion of abnormal SNAP in a sural-sparing pattern improves EDX of GBS. The increase in sensitivity is due to simplification of the MNC criteria for the non-MFS group. In these patients, the sural-sparing pattern did not increase the yield of the stricter traditional criteria. However, in the MFS group where the MNC abnormalities are subtle relative to SNAP changes, the sural-sparing pattern did contribute to sensitivity. Nine MFS patients with the sural-sparing pattern had normal motor NCS based on traditional EDX criteria. The addition of sural-sparing and using simplified MNC criteria allowed 3 of these cases to be labeled as “Definite GBS” and a further 4 to be labeled as “Probable GBS”. The remaining 2 had normal motor NCS despite the simplified criteria; the presence of sural-sparing allowed them to be labelled as “possible GBS”.

We believe the “Probable” and “Possible” categories reflect the common clinical reality of EDX uncertainty in many cases of GBS, also factored in the Brighton criteria. Due diligence should be given to rule out co-morbid conditions such as diabetic neuropathy, carpal tunnel syndrome and age-related decrease in SNAP when applying these criteria. In the “Probable” category, substantially more corroborative clinical and laboratory findings are required compared to those who fulfil “Definite” criteria. In the “Possible” category, the NCS findings are not inconsistent with GBS, but the onus of proof lies largely on clinical and laboratory findings such as cytoalbuminergic dissociation and specific anti-ganglioside antibodies such as anti-GQ1b antibodies. We admit that the derivation of the above categories is quite arbitrary. Many GBS patients at point of presentation, when a diagnostic EDX is perhaps most useful to the clinician, have relatively mild and non-specific nerve conduction abnormalities; especially those with the non-demyelinating subtypes of GBS and MFS. Hence, the criteria in each category are quite broad. Besides the isolated sural SNAP-involving pattern, we have not found any other sensory pattern that could reasonably be excluded from GBS EDX criteria. Furthermore, we did not have a control group. Our study is based on consecutive patients recruited prospectively into a GBS patient database at a single centre only. Patients initially diagnosed to have GBS but subsequently had an alternate diagnosis were removed. Therefore, we did not have a prospectively collected control cohort to apply our new EDX criterion on. Its utility, both in terms of sensitivity and in particular specificity, has to be verified and validated by prospectively applying it to a set of patients presenting with acute flaccid paralysis. We are currently planning such a study on cohorts of GBS patients from different centres and countries.

The weakness of sensory NCS over motor NCS in the EDX of GBS is that SNAP abnormalities take a longer time to appear. Albers *et al*. showed that the nadir of motor conduction abnormalities occur in the 3rd week of illness, while sensory changes peak at 4th week^5^. This would impact on the utility of early EDX studies, when GBS diagnosis may be arguably more crucial. Sensory studies are also not useful in differentiating axonal and demyelinating subtypes. SNAP amplitude readily decreases in both pathologies. However, there is a need to shift the focus away from delineating demyelinating and axonal pathology in the initial EDX, a task possibly best served by serial studies; and adopt a simplified GBS EDX criterion that lends a graded level of confidence to the clinician facing a patient with acute flaccid paralysis.

## Methods

We analysed the records of consecutive GBS patients that were prospectively databased at a tertiary neurology centre in Singapore. The methodology of the database has been previously published^10^. The diagnosis of GBS and MFS subtype was based on Brighton criteria and the patients were followed till near-complete recovery to ensure veracity of diagnosis. In addition, we sought corroborative evidence for diagnosis of MFS and MFS-GBS overlap cases by testing for raised anti-GQ1b antibodies. We excluded patients with pre-existent peripheral neuropathy including diabetic neuropathy. Information on co-existent diseases were collected, and corroborated by clinical and laboratory evaluation at the point of recruitment.

The initial NCS was used for analyses, and was performed according to protocols previously published^10^. NCS was done on one upper and one lower limb, consisting of median, ulnar, tibial and peroneal motor nerve studies; and median, ulnar and sural sensory nerve studies. Sensory nerve recordings were anti-dromic. Motor and sensory NCS abnormalities were defined as listed below, using age and height adjusted norms that were derived from 245 normal controls^10^:

- Any CMAP or conduction velocity values below the lower limit of normal thresholds based on normal control data,
- Any DML prolongation or F wave abnormalities exceeding the upper limit of normal thresholds based on normal control data,
- Any temporal dispersion with increased duration more than 30%,
- Any conduction block (decrease of more than 50% in proximal CMAP compared to distal without an increase on duration of more than 30%).
- SNAP amplitude below the lower limit of normal for age and height. The many technical issues and potential confounding from co-existent disorders prompted us to omit distal sensory latency.
- We defined sural-sparing as a greater percentage decrease from normal of the median and or ulnar SNAP amplitude compared to that of the sural SNAP:

$$\begin{array}{c}\frac{({\rm{Normal}}\,{\rm{Median}}\,{\rm{or}}\,{\rm{Ulnar}}\,{\rm{SNAP}})\,\mbox{--}\,(\mathrm{Patient}\mbox{'}s\,{\rm{Median}}\,{\rm{or}}\,{\rm{Ulnar}}\,{\rm{SNAP}})}{({\rm{Normal}}\,{\rm{Median}}\,{\rm{or}}\,{\rm{Ulnar}}\,{\rm{SNAP}})}\\  > \\ \frac{{\rm{Normal}}\,{\rm{Sural}}\,{\rm{SNAP}}-\mathrm{Patient}\mbox{'}s\,{\rm{Sural}}\,{\rm{SNAP}}}{{\rm{Normal}}\,{\rm{Sural}}\,{\rm{SNAP}}}\end{array}$$

The normal SNAP amplitudes were based on age and height adjusted norms^12^.

Using serial NCS, we have previously shown that the above formula, which is based on percentage decrease, is more sensitive at detecting relative sural-sparing than one that relies on comparing absent or abnormal upper limb SNAPs against normal sural SNAP^10,12^. The NCS was done on one side, and the sural-sparing calculation was done based on these nerves.

We applied the following EDX criterion to the initial NCS of a cohort of patients proven to have GBS on prolonged follow-up (Table 1). Definite GBS: two abnormal motor nerves and the sural-sparing pattern of sensory abnormality. Probable GBS: either (i) one abnormal nerve and the sural-sparing pattern of sensory abnormality or (ii) two abnormal motor nerves with either a normal sensory nerve NCS or a decrease in SNAP in diffuse non sural-sparing pattern (that is a similar degree of decrease in upper and lower limb SNAPs). Possible GBS: (i) one abnormal motor nerve with or without sensory nerve abnormalities, or (ii) normal motor NCS with either diffuse sensory nerve abnormalities or the sural-sparing pattern of sensory abnormality. Isolated sural involvement is rare in GBS. Albers *et al*. studied 70 patients and found no patient with this pattern^5^. Hence, for the probable and possible categories we stipulated that the abnormality on sensory nerve could not be isolated to the sural nerve. We compared the diagnostic yield of the above criterion against GBS EDX diagnosis based on the criteria described by Ho *et al*.^2^ and Hadden *et al*.^3^ Patients whose NCS were abnormal by either criteria (regardless of classification into any of the four categories of primary demyelinating, primary axonal, inexcitable and equivocal) were deemed to have satisfied “traditional EDX criteria” for GBS. No attempt was made to differentiate axonal and demyelinating subtypes.

The institutional review board (Singhealth Centralised Institutional Review Board, Singapore) approved the database and related studies. All relevant guidelines and regulations were adhered to strictly. All patients gave informed consent.

Data published in this article is available for review on request.
