# Publisher Correction: A Simplified, Graded, Electrodiagnostic Criterion for Guillain-Barré Syndrome That Incorporates Sensory Nerve Conduction Studies

**DOI:** 10.1038/s41598-020-57578-7

**Published:** 2020-01-14

**Authors:** Thirugnanam Umapathi, Christen Sheng Jie Lim, Brandon Chin Jie Ng, Eunice Jin Hui Goh, O. Ohnmar

**Affiliations:** 1 National Neuroscience Institute, Department of Neurology, Singapore, Singapore; 2 National University Hospital, University Medicine Cluster, Singapore, Singapore; 3 National University of Singapore, Yong Loo Lin School of Medicine, Singapore, Singapore; 4 Yangon General Hospital, Yangon, Myanmar

Correction to: *Scientific Reports* 10.1038/s41598-019-44090-w, published online 22 May 2019

This Article contains a typographical error in the Results section under subheading ‘Application of modified GBS EDX criterion to the cohort’ where,

“When using our proposed GBS EDX criterion, 39 (35.8%), 47 (43.1%) and 13 (11.9%) of all GBS patients were classified as “Definite”, “Probable” and “Possible” GBS respectively (total 96.6%; Table 2).”

should read:

“When using our proposed GBS EDX criterion, 39 (35.8%), 47 (43.1%) and 13 (11.9%) of all GBS patients were classified as “Definite”, “Probable” and “Possible” GBS respectively (total 90.8%; Table 2).”

